# HPV Vaccination Uptake and Acceptability of HPV/HIV Integrated Services Models for Adolescent Girls in Mozambique and Zimbabwe: The AIM-HPV Implementation Research Study

**DOI:** 10.3390/vaccines14060503

**Published:** 2026-06-03

**Authors:** Michelle M. Gill, Assucênio Chissaque, Edna Viegas, Lillian Chinyanganya, Hilda Bara, Lauren Greenberg, Nontokozo Gava, Mahira Amade, Bridget Kanengoni, Angela Mushavi, Leonildo Augusto Nhampossa, Aleny Couto, Neiva Banze, Humberto Inguane, Epifânia Orlando Raimundo, Patricia Pérez Martin, Laura Guay, Rhoderick Machekano

**Affiliations:** 1 Elizabeth Glaser Pediatric AIDS Foundation, Washington, DC 20036, USA; lgreenberg@pedaids.org (L.G.); lguay@pedaids.org (L.G.); rmrodmach@gmail.com (R.M.); 2 Elizabeth Glaser Pediatric AIDS Foundation, Maputo 1100, Mozambique; assucenyoo@gmail.com (A.C.); humbingua@gmail.com (H.I.); patriciaperez7@gmail.com (P.P.M.); 3 Instituto Nacional De Saúde (INS), Maputo 1120, Mozambique; edna.viegas@ins.gov.mz (E.V.); mahira.amade@hotmail.com (M.A.); neiva.banze@ins.gov.mz (N.B.); epifaniaamome@gmail.com (E.O.R.); 4 Elizabeth Glaser Pediatric AIDS Foundation, Harare P.O. Box CY 1122, Zimbabwe; lchinyanganya@gmail.com (L.C.); bara578ht@gmail.com (H.B.); nontokozogava@gmail.com (N.G.); bridgetkanengoni7@gmail.com (B.K.); 5 Zimbabwe Ministry of Health and Child Care, Harare P.O. Box CY 1122, Zimbabwe; mushavia@yahoo.co.uk; 6 Mozambique Ministry of Health, Maputo 1100, Mozambique; leonhamp05@gmail.com (L.A.N.); coutoaleny@gmail.com (A.C.); 7 Milken Institute School of Public Health, George Washington University, Washington, DC 20052, USA

**Keywords:** HIV, HPV, vaccination, integration, adolescents, Mozambique, Zimbabwe

## Abstract

Background/Objectives: Girls living with HIV (GLHIV) or vulnerable to HIV have a higher risk of HPV infection and cervical cancer as they age. We determined acceptability and vaccination uptake after integrating HPV vaccination into HIV prevention and treatment services for girls in Mozambique and Zimbabwe. Methods: Pre-integration and integration HPV vaccination information were abstracted from routine records of girls aged 9–14 years offered HPV vaccine through HIV services in 54 health facilities (HFs) and surrounding communities between February and December 2025. Caregivers participated in quantitative surveys about vaccine perceptions and integration model experiences in a subset of 16 HFs. Results: In total, 6377 records of girls (median age: 11 years) were abstracted. Among the vaccine recipients, 63 (3.0%) girls received vaccine pre-integration and 2019 (97.0%) post-integration in Mozambique and 743 (17.3%) pre-integration and 3541 (82.7%) post-integration in Zimbabwe. Among GLHIV, 95.8% and 69.6% received a first HPV vaccine in Zimbabwe and Mozambique, respectively. Full vaccination with two doses occurred in 49.1% of eligible girls in Mozambique and 73.9% in Zimbabwe. Overall, 461 (67.8%) caregivers had heard of the HPV vaccine and 85.9% of cervical cancer, 99.6% were satisfied with vaccination in integration settings, and 78.6% preferred facility-based vaccination models. Conclusions: We demonstrated that HPV/HIV service integration was an effective strategy to increase HPV vaccine uptake among young girls at increased risk of HPV and cervical cancer. We found high vaccine and model acceptability and awareness of cervical cancer among caregivers. Optimization of this approach requires better integrated tools and model adaptations to fit the needs of girls and health systems.

## 1. Introduction

Cervical cancer is the fourth most common cancer globally, with 660,000 cases estimated in 2022, disproportionately affecting sub-Saharan Africa [1,2]. Cervical cancer is a leading cause of cancer deaths among women in Africa, particularly among women living with HIV [1,2]. Persistent infection with the Human Papillomavirus (HPV), an asymptomatic sexually transmitted infection (STI) that many sexually active individuals acquire in their lifetime, may lead to the development of cervical cancer [3]. While the majority of infections with HPV are cleared naturally by the immune system, some (<10%) HPV infections may persist and induce cellular dysplasia and neoplasia that can lead to cancer [4]. Co-infection with HIV leads to a higher risk of persistent HPV infection and progression to dysplasia, increasing the risk of developing cervical cancer nearly six-fold [5]. The intersection of the HIV and HPV epidemics in southern Africa has resulted in an estimated 40% of all cervical cancer cases that are attributable to HIV in this geographic area [2].

The availability of effective HPV vaccines has led to a significant decrease in the incidence of cervical cancer in settings with successful implementation of vaccination programs, demonstrating that cervical cancer is preventable. However, implementation of national HPV vaccination programs and resulting vaccine coverage remain suboptimal in many low- and middle-income countries where the incidence of cervical cancer and HIV infection is the highest. In 2020, the World Health Organization (WHO) released a global strategy for the elimination of cervical cancer that includes a target of 90% of girls fully vaccinated against HPV by age 15 years [6]. This highlights an urgent need to achieve and sustain high HPV vaccination coverage among all adolescent girls and young women, but particularly girls living with HIV (GLHIV) and those at increased risk of acquiring HIV, to reduce the dual burden of HIV and HPV-related disease.

In 2022, to facilitate a more feasible and cost-effective strategy for the incorporation of HPV vaccination into national programs, WHO revised the recommended vaccination schedule to include an option for a single dose of the vaccine to girls without HIV infection and a two-dose schedule (6-month interval) for GLHIV from the previously recommended two- and three-dose schedules, respectively [7]. Countries with the highest burden of HPV infections are in various stages of implementing HPV vaccination, including incorporating the revised immunization schedule into the national program and identifying the most effective strategies to reach the most girls in need. Current delivery strategies include school-based, community outreach, or targeted clinic-based campaigns, each of which has its own strengths and limitations. For example, school-based models may have high operational costs and limited reach for marginalized populations, such as out-of-school girls [8,9].

Mozambique and Zimbabwe are both low to middle-income countries in sub-Saharan Africa with high burdens of cervical cancer and HIV and persistent challenges in accomplishing adequate HPV vaccination coverage. In 2022, cervical cancer accounted for 33.4% of new cancer cases in females in Mozambique and 31.4% in Zimbabwe [10]. Mozambique (2021) and Zimbabwe (2018) introduced the HPV vaccine in their vaccination programs through their Expanded Programs on Immunization (EPI), targeting mainly 10-year-old girls and girls in grades 5 and 6 (or 10 and 11-year-olds if out of school [OOS]), respectively, via school and health facility delivery [11,12].

In Mozambique, between 2020 and 2023, a study found a 31.3% prevalence of HPV among all women, with the highest prevalence in women living with HIV (39.7%) compared to those without HIV infection (24.3%) [13]. National vaccination coverage of 68% for the first dose and 56% for the second dose was achieved in 2023 [14]. In Zimbabwe, approximately 1900 women die of cervical cancer each year, the highest cancer-related mortality in the country [15]. In 2024, the vaccination coverage was about 80% of eligible girls for the first dose and 51% for the second dose, as reported to the WHO [16]. Mozambique and Zimbabwe adopted the WHO modified recommendations, maintaining the two-dose vaccine schedule for populations living with HIV but including a one-dose vaccine schedule for all others in 2024 and 2025, respectively. In October 2025, a week-long, national multi-age cohort campaign for HPV vaccination was rolled out in Mozambique for girls aged 12–18 years [17].

Given the intersection of HIV and HPV infections, there is a critical need to ensure that girls living with, or at risk of HIV acquisition, receive the appropriate HPV vaccination. We conducted an implementation research study to determine the acceptability and effectiveness of integrating HPV vaccination into HIV prevention and care and treatment (C&T) services for girls in Mozambique and Zimbabwe.

## 2. Materials and Methods

### 2.1. Study Design

The “Assessing HIV Integrated Service Models for HPV Vaccination Among Adolescent Girls” (AIM-HPV) study was a Type 2 hybrid effectiveness implementation research study with a mixed-methods, quasi-experimental, pre-post study design. In this type, “an implementation intervention/strategy of some kind is being tested alongside and in support of a clinical intervention under study” [18]. These designs include both clinical effectiveness and implementation outcomes. The study was conducted in Zimbabwe and Mozambique between February and December 2025 and included chart abstraction of routine service data and cross-sectional quantitative surveys to evaluate the HPV/HIV vaccination integrated model. The study was based on the Practical, Robust Implementation and Sustainability Model (PRISM) and explored patient and organizational characteristics and perspectives on the HPV integration into HIV services intervention [19]. Outcome measures were based on the Reach, Effectiveness, Adoption, Implementation, and Maintenance (RE-AIM) framework [20].

### 2.2. Study Setting

The study was conducted in Gaza and Inhambane provinces in Mozambique, representing regions with high and medium HIV prevalence in the country, respectively [21]. Two districts in Gaza Province (Chongoene and Limpopo) and one district in Inhambane Province (Maxixe) were selected for the study based on the presence of the United States President’s Emergency Plan for AIDS Relief (PEPFAR)-supported Determined, Resilient, Empowered, AIDS-free, Mentored and Safe (DREAMS) HIV prevention programs. Initially, 24 health facilities (HFs) and surrounding communities with a DREAMS program in these three districts were included in the study as HPV/HIV integration sites, implementing a co-delivery or a mix of co-delivery and co-location approaches. *Co-delivery* was defined as the same provider delivering HIV-related services and HPV vaccination. *Co-location* was defined as HIV-related services provided at the same site around the same time as HPV vaccination, though the provider and department may differ. Six additional HFs in five other districts in Inhambane were purposively selected for their high volume of girls aged 9–14 years to implement a co-location model only for girls receiving HIV C&T. Two co-delivery HFs in Gaza were later recategorized as co-location sites due to implementation model adaptations, resulting in 22 co-delivery or mixed co-delivery/co-location HFs and eight co-location-only HFs.

In Zimbabwe, the study was conducted in the Mazowe district of Mashonaland Central province and the Harare district of Harare Metropolitan province. While HIV prevalence is similar [22], these districts represented distinct rural and urban settings and programs that reached different populations of beneficiaries: Orphans and Vulnerable Children (OVC) programs in Harare and DREAMS programs in Mazowe. Twenty-four HFs and their surrounding communities were selected for the study. In Mazowe, this included 14 facilities with the highest numbers of GLHIV 10–14 years in the rural and farming areas of the district (out of 38 HF). In Harare, all ten HFs that provided OVC services were selected out of 53 HFs in the district.

### 2.3. Intervention Description

Existing HIV prevention, C&T services reach a vulnerable population of 9–14-year-old girls who are at risk of acquisition of HPV infection and development of cervical cancer and HIV infection as they age. Comprehensive health, education, psychosocial support, caregiver support and community linkages are in place through these services. Since these girls are regularly in contact with health and community services, the intervention integrated a two- dose HPV vaccination regimen for GLHIV into HIV treatment services, and a single HPV vaccine for all other girls receiving HIV prevention services. The goal was to impart a minimal burden to both the system and the young girls and provide a cost-efficient method of ensuring that this high-priority population would be reached with HPV vaccination. A Stop Work Order (SWO) was issued in January 2025 for PEPFAR which affected funding to HIV prevention programming to adolescent girls and the extent to which DREAMS/OVC services could be leveraged for HPV vaccination.

**Figure 1:**
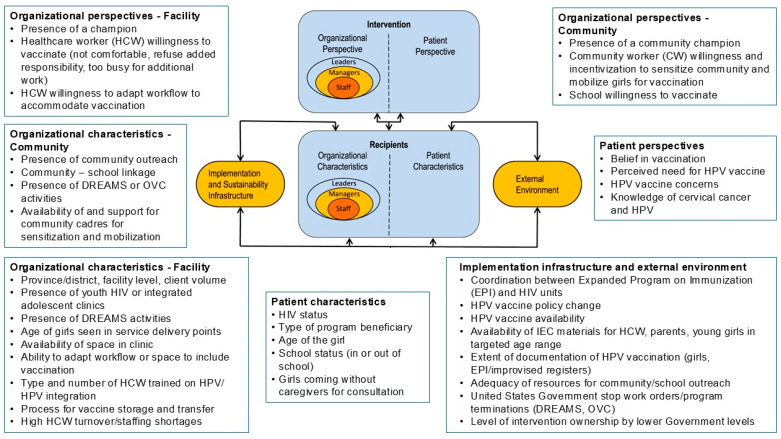
HPV/HIV Vaccination Service Integration Framework for the Design of Implementation Models According to PRISM Domains [23].

The overall intervention framework, informed by PRISM elements (Figure 1), was determined through multiple stakeholder engagements, including input from youth advisory committees in each country. Specific implementation models of HPV/HIV service integration were then designed and later adapted at the country and HF levels based on the framework. Some elements were similarly implemented across both countries, such as adaptation of information, education, and communication (IEC) materials and community and health provider sensitization and training. 

*Mozambique Model.* Integration in HFs occurred at the HIV clinic during ART refill visits or in Adolescent and Youth Friendly Services (in Portuguese, Serviços Amigos dos Adolescentes e Jovens [SAAJ]) clinics. Twenty-two study HFs had SAAJ clinics which provide comprehensive services for adolescents with and without HIV starting at age ten years, including HIV and STI screening, counseling and treatment, contraception, and post-violence care. HF vaccination points varied by PRISM domains, organizational perspectives and characteristics, such as the presence of SAAJ clinics, space and provider willingness at HIV clinics, and presence of DREAMS activities. Site-level models involved one or two of the following approaches: (1) girls received C&T services and HPV vaccination at the HIV clinic; (2) girls received C&T services at the HIV clinic with intra-facility linkage to EPI for vaccination; (3) girls received C&T services at the HIV clinic with intra-facility linkage to SAAJ for vaccination; and (4) girls received C&T or preventative services and HPV vaccination at SAAJ clinic. Additionally, in four HF, mobile brigades were organized by SAAJ to deliver HPV vaccination alongside HIV prevention and other health services in communities. At the eight co-location-only HF, GLHIV receiving C&T services at the HIV or SAAJ clinic only were referred to EPI for vaccination. For any model where girls were not vaccinated at the same point of service delivery, they were provided with a referral coupon, escorted by a provider, or directed to reach the vaccination point on their own.

Given SWO funding restrictions, community-based DREAMS services were suspended in Gaza Province prior to model introduction. DREAMS clinical activities, such as educational sessions on sexual and reproductive health, continued at all HFs with SAAJ and were leveraged for demand creation and referral of vulnerable girls for HPV vaccination. In Inhambane Province, the standard DREAMS package was provided throughout most of the intervention period in the five co-delivery or mixed co-delivery/co-location HF. DREAMS mentors leveraged facility- and limited community-based activities to generate demand for HPV vaccination. In both provinces, DREAMS beneficiaries were most often reached with vaccination through SAAJ-run services. All DREAMS activities were terminated at the end of September 2025.

Caregivers provided verbal consent for HPV vaccination for girls aged 9–12 years when accompanying them to the facility or community vaccination point. Girls aged 13–14 years could consent themselves. The provider at the vaccination entry point (HIV, SAAJ) was responsible for completing all relevant tools to document vaccination, including the handheld vaccination card, vaccination register and HIV records.

*Zimbabwe Model.* The initial intervention design at most sites (*n* = 17) was a combination of HPV vaccination co-delivery at the HIV clinic and co-location with C&T services at the HIV clinic, with intra-facility linkage for HPV vaccination to EPI or a family and child health (FCH) clinic for GLHIV. However, most sites (*n* = 22) shifted to a primarily co-location model, in that girls from the HIV clinic would be escorted by the HIV clinic nurse or lay worker to the EPI, FCH clinic, or outpatient department (OPD) (in two sites) for HPV vaccination. Girls were also vaccinated by non-HIV clinic nurses attending regular antiretroviral (ART) adolescent support group meetings on Saturdays. Shifts were informed by organizational perspectives and characteristics, such as vaccine storage and handling processes, and clinic flow adaptations to reduce wait times in the HIV clinic and improve overall HF efficiency, especially those with nursing staff shortages. Two small sites with most services already integrated vaccinated girls at the HIV clinic. Generally, documentation in the HPV register was done by the nurse administering the vaccine while the HIV nurse documented vaccination in HIV-specific records.

Peer leaders and community workers (CWs) supported nurses in HPV health education, demand generation, and tracking vaccine-eligible girls, including home visits. CWs also conducted door-to-door health education sessions on HPV, designed to ensure OVC and DREAMS beneficiaries were reached but included all girls aged 9–14 years. These beneficiaries who presented at the study HFs were vaccinated for HPV in the EPI/FCH clinic or OPD. HPV vaccination for DREAMS beneficiaries in Mazowe shifted in March 2025 primarily from HFs to schools, due to concerns that some girls could not travel the long distances to study HFs. Sensitization, demand generation and mobilization for vaccination then shifted to school health staff. They maintained DREAMS registers used to identify girls eligible for vaccination. Some OVC in Harare were also vaccinated through schools where there was already planned tetanus/diphtheria vaccination, and communities. While funding restrictions limited DREAMS and OVC programming, the AIM-HPV project supported healthcare worker (HCW) lunch allowances and transport for community and school outreach so that beneficiaries could still be reached for vaccination. Caregiver verbal consent for vaccination was often communicated through their children, community cadres involved in mobilization, or school health staff; if not, facility staff sought caregiver consent via phone.

### 2.4. Study Population, Sampling and Recruitment

*Chart Abstraction of Service Data.* Girls living with HIV or at risk of infection were offered the HPV vaccine through multiple service entry points. They were first screened to determine if they had received the HPV vaccine previously, usually by self-report, as documentation was typically not available. Number and dates of any pre-integration and integration HPV doses were recorded in routine clinic health records including HPV vaccine/EPI registers and HIV clinic records. Records were abstracted from girls aged 9–14 years who attended HIV prevention and C&T services or were reached through DREAMS and OVC platforms and screened for HPV vaccination eligibility in all study HF and surrounding communities to determine HPV vaccine uptake.

*Caregiver Survey.* Parents or primary caregivers (≥18 years) of girls 9–14 years old who attended these services and were eligible for HPV vaccination were recruited to take part in quantitative surveys in a subset of eight study HFs in each country. This subset was purposively selected to represent different service delivery models, a mix of HF levels, and districts. Acceptability of vaccine integration was defined as the proportion of caregivers satisfied with and willing to recommend HPV vaccination. A target of 385 caregivers in each country, split equally between those with girls with and without HIV, was estimated to be sufficient to determine acceptability of 50% or more.

HCWs and peer and community cadres working at a study facility or an outreach setting typically referred potentially eligible caregivers who were interested in hearing about the study to research assistants (RAs), research nurses trained on the protocol and research ethics. In cases where the girl was vaccinated but unaccompanied by her caregiver, girls themselves were encouraged to invite the caregiver to the HF in Mozambique. In Zimbabwe, potential caregiver participants were encouraged to return to the HF by health or community providers. In both countries, RAs also worked with site staff to coordinate ahead of scheduled visits to maximize the availability of potential participants. RAs attempted to recruit caregivers who refused HPV vaccination for their child; however, there was only one such caregiver in Zimbabwe.

### 2.5. Data Collection

*Chart Abstractions.* Any documented vaccinations among girls in our study population prior to the intervention period were captured from May (Zimbabwe) and June (Mozambique) 2021 to March 2025. The integration period began in the first HF in Zimbabwe in October 2024 and the first HF in Mozambique in March 2025 to December 2025. The overall pre-integration and integration periods overlap since individual HFs varied in intervention introduction timing. Following vaccine integration, HPV vaccination and selected HIV program data were abstracted by RAs from paper-based, standard and modified national and facility and community-level tools from 28 February to 10 December in Zimbabwe and 1 April to 5 December 2025 in Mozambique.

Demographic data (e.g., age, beneficiary group), HIV status, and HPV vaccination information (e.g., doses, location, dates) were obtained from source documentation. Girls included were those who had been vaccinated before the introduction of the integration model to the extent possible, girls who had declined HPV vaccination, and girls receiving one or two doses of HPV as part of the integrated model. There was poor documentation and linkage across different sources of previous vaccinations among potentially eligible girls. To address this issue in Zimbabwe, names of C&T, DREAMS, and OVC beneficiaries in our study sample were abstracted into a temporary electronic file and cross-referenced with names (and dates of birth as needed for confirmation) from existing vaccination lists from study HFs and schools in facility catchment areas that were manually entered into an electronic file. All files were maintained securely and separately from the study database with limited access. This file was deleted immediately after the study data had been collected and verified.

*Caregiver Surveys.* Surveys were conducted between 28 February and 3 October 2025 in Zimbabwe and between 4 April and 17 October 2025 in Mozambique. Surveys took place when caregivers accompanied their child to study HFs or as part of community vaccination activities, where their child was offered the first or second dose of the HPV vaccine, or at another time convenient to the caregiver. Each caregiver who participated in the survey received transport reimbursement: 200 Meticais (equivalent to about three United States [US] dollars in Mozambique) and US$5 in Zimbabwe. Depending on preference, surveys were conducted in Shona for most participants and in English for a few caregivers in Zimbabwe. All surveys in Mozambique were conducted in Portuguese. Information collected included caregiver background and demographic characteristics, general beliefs and attitudes toward vaccination, knowledge and perceptions of HPV infection and HPV vaccination, prior vaccination experiences, and factors influencing acceptance or refusal of HPV vaccination for their daughters. If caregivers had multiple girls aged 9–14 years with an eligible beneficiary status in their care, child-specific information was captured for two girls.

All data were entered into electronic, pre-tested forms with built-in logic and quality checks and synced to a study-specific REDCap database; data were encrypted in transit. Each participant was assigned a unique study code, and all identifiers were stored separately from research data. Participant names were not recorded on study instruments and no personal identifiers were collected in the study database. REDCap was hosted on a secure, authenticated cloud server. The data manager ran regular programmed quality checks, issued queries, and produced monitoring reports for quality assurance.

### 2.6. Data Analysis

Service delivery characteristics of girls by HIV status and caregiver survey data and demographics were described using descriptive statistics: frequencies and proportions to summarize categorical variables and medians (interquartile ranges [IQR]) for continuous variables. Selected caregiver survey data were also summarized by caregivers of girls with and without HIV.

In Zimbabwe, the denominator for HPV vaccination coverage was defined as all GLHIV receiving C&T services who were eligible at any time point during the integration period based on study HF reporting. This included girls who aged in or out, any transfers in or out of the study HFs, and girls identified as HIV-positive at any point during the study. The number of DREAMS beneficiaries included all girls who were registered in a facility catchment area and was determined primarily through DREAMS records maintained at schools. The OVC denominator was based on program enrollment from 2024. Due to a lack of access to DREAMS/OVC records (which included HIV status) maintained by partners whose work under PEPFAR was terminated, most beneficiaries could only be categorized as having an unknown HIV status for the study. While DREAMS beneficiaries were primarily HIV-negative, about 80% of OVC had been previously estimated to be HIV positive [24].

In Mozambique, the electronic ART database maintained at study HFs was used to determine the number of girls aged 9–14 years with HIV (denominator for the HIV clinic combined with SAAJ). As this only provided monthly counts, we selected the month between April and November 2025 with the highest number per site to best capture the total number of vaccine-eligible girls. For girls without HIV or of unknown HIV status, the denominator was determined by register counts of all girls attending their first SAAJ consultation (in the clinic or community) in which the package of SAAJ services was to be offered, including HPV vaccination. At two HFs with register issues, we used the numbers reported to the Ministry of Health (MoH). Because of the SWO, there were limited DREAMS staff to support vaccination documentation, and the routine records did not distinguish the subset of SAAJ girls who were also DREAMS beneficiaries.

To calculate ≥1 dose coverage, girls in the study population were included in the numerator if they received one or two doses of vaccine during the pre-intervention and/or intervention periods. Second dose coverage for all GLHIV was calculated using the number of girls who had received a second dose during the intervention period among girls eligible for a second dose. It includes GLHIV who received both doses during the intervention period or one dose pre-intervention and one dose during intervention. Coverage is described by program beneficiary type and country.

Mixed effects bivariable logistic regression models were used to identify facility (e.g., level, volume, district) and individual (e.g., age, program beneficiary status) characteristics potentially associated with full HPV vaccination coverage, defined as both doses received under the intervention models, among GLHIV who were eligible to receive a second dose by the end of the study, accounting for potential facility-level clustering from routine service data. Only in Mozambique could the type of model (co-delivery or co-location) be assessed, given that most HFs in Zimbabwe implemented a similar mix of approaches. Characteristics significantly associated with vaccination at *p* ≤ 0.1 are included in multivariable models. Adjusted odds ratios and associated 95% confidence intervals were used to identify and quantify characteristics independently associated with HPV vaccination. All analyses were performed using Stata version 19 (StataCorp LLC, College Station, TX, USA), R 2025 (R Foundation for Statistical Computing, Vienna, Austria), and RStudio 2024 (Posit Software, PBC, Boston, MA, USA).

## 3. Results

### 3.1. Chart Abstractions

Overall, records of 6377 girls were abstracted across both countries. Girls were a median age of 11 years (IQR 10–13), with 10.9% (*n* = 695) < 10 years of age; 0.7% of girls (*n* = 44) were outside of the eligibility age range (either 8 or 15 years of age). GLHIV comprised 17.1% (*n* = 1093) of the study population; 66.0% were in Mozambique. The majority (86.4%) of girls in Zimbabwe had an unknown HIV status; only 0.7% were not in school, and 1.1% had an unknown school status (data not available in Mozambique).

In Mozambique, 2082 (65.0%) girls received the HPV vaccine, of which only 63 (3.0%) had documentation of a vaccination prior to the HPV/HIV intervention, and 2019 (97.0%) received ≥1 doses during the intervention period (Table 1). There were no documented refusals. For dose 1, 1570 (77.8%) girls were vaccinated in HFs, 84 (4.2%) as part of DREAMS community outreach, and 364 (18.0%) through mobile brigades (one missing location). No GLHIV had received two doses prior to integration.

At the end of data collection, there were 320 GLHIV who were eligible to receive their second dose and 401 who had not yet reached six months post-initial dose. Of the 157 (49.1%) eligible girls who received their second dose, 102 girls received both doses under integration and 55 received their first dose pre-integration and their second dose during integration. For the 163 (50.9%) eligible girls who were not vaccinated, it was <8 months since their first dose.

In Zimbabwe, 4284 (96.6%) girls received the HPV vaccine, of which 743 (17.3%) had documentation of vaccination prior to HPV/HIV integration and 3541 (82.7%) received ≥1 dose during integration (Table 2). Coverage rates were above 95% for all beneficiary groups. There were 11 refusals; six were GLHIV. For girls in C&T, reasons for refusal included being too busy with farming, the caregiver being uncomfortable with vaccination, failure to get consent from the primary caregiver, the child being lost to follow-up, and religious beliefs. For dose 1, 1055 (29.8%) girls were vaccinated in HFs, 865 (24.5%) as part of community outreach, and 1616 (45.7%) through schools (five missing locations). Some DREAMS/OVC beneficiaries (*n* = 415) of HIV-negative or unknown status received a second HPV dose during integration, and four received two doses prior to the study period, as the policy in the country until late April 2025 was to vaccinate all girls with two doses.

At the end of the study, 306 GLHIV were eligible to receive their second dose and 47 had not yet reached six months post-initial dose. 226 (73.9%) had received their second dose: 189 received both doses under integration and 37 received their first dose pre-integration and their second dose during integration. Among the 80 girls who were eligible but did not receive the second dose, it had been 6–8 months for 59 girls (73.8%) and >8 months for 21 girls (26.2%) since their first dose by the end of the study.

In Zimbabwe, there were no individual or facility-level factors associated with receiving a second dose, but the model was limited by the variables available for all girls in routine clinic records (Supplementary Table S1). In Mozambique, a girl was more likely to receive a second dose at a co-location-only site compared to an integration site (44.6% vs. 36.9%, *p* = 0.002) but significantly less likely to receive a second dose in Inhambane, where six of the eight co-location-only sites were located, compared to Gaza Province (27.6% vs. 49.0%, *p* < 0.001, Table 3).

### 3.2. Caregiver Survey

In total, 668 caregivers who were offered HPV vaccination for children in their care contributed to survey findings: 385 in Zimbabwe, 283 in Mozambique. In Mozambique, 290 caregivers were interviewed for the study, but seven from one HF were excluded because the signed informed consent forms could not be located. Overall, 93.7% participants were female with a median age of 39 years (IQR 32–47). Caregivers in Mozambique were more likely to have received no formal education or attended primary school only compared to Zimbabwe (89.4% vs. 47.8%, *p* < 0.001). About half of Zimbabwe caregivers (51.4%) had completed high/secondary school or higher. Across both countries, 42.5% (*n* = 284) of caregivers were living with HIV. Overall, 7.3% (*n* = 49) of caregivers had more than one vaccine-eligible child in their care for a total of 700 children; 42.4% of children (*n* = 311) were living with HIV (Table 4). Most GLHIV came from the HIV clinic; however, 8.0% of them were recruited from SAAJ in Mozambique and 3.2% from DREAMS or OVC services. Few girls overall (2.6%) were OOS.

Table 5 describes knowledge and perceptions related to HPV and cancer that may influence uptake and acceptability of the HPV vaccine and integration. Most caregiver participants (85.9%) had heard of cervical cancer, with 35.4% of those caregivers reporting knowing someone with cervical cancer. Knowledge of cervical cancer (74.2% vs. 94.5%), HPV (45.1% vs. 64.7%) and the HPV vaccine (49.8% vs. 82.1%) were lower in Mozambique compared to Zimbabwe (*p* < 0.001). Overall, more caregivers had heard of the HPV vaccine (67.8%) compared to HPV (56.2%) in both countries, but this difference was considerably larger in Zimbabwe (82.1% vs. 64.7%). In both countries, more caregivers of GLHIV knew about HPV and the vaccine, but only in Mozambique were these caregivers also more aware of cervical cancer (Supplementary Table S2). Of those who had heard of the vaccine, most (85.3%) had no concerns with it. Concerns included the vaccine’s effect on fertility, feeling sick after, and pain at the injection site. Caregivers overwhelmingly preferred HPV vaccination information from HCWs (87.9%). In Zimbabwe, caregivers of GLHIV had a greater preference for vaccination messaging delivered through TV and radio than caregivers of girls without HIV (78.3% vs. 33.2%, Supplementary Table S2).

Only one biological mother of a 13-year-old with HIV refused the vaccine with no reason provided. Over three-quarters of caregivers (78.5%) agreed to vaccination because they believed it would protect against HPV/cancer.

The majority of caregivers in both countries preferred HPV vaccination delivery at HFs. More caregivers favored vaccination as part of other services over vaccination at immunization clinics in Zimbabwe (54.0% vs. 19.8%); this preference for integrated delivery increased to 75.5% among caregivers of GLHIV compared to 38.8% among caregivers of girls without HIV. In contrast, slightly more caregivers in Mozambique preferred immunization clinics (44.2% vs. 40.9%); preference for the immunization clinic increased to 46.7% among caregivers of GLHIV versus 41.7% among those without HIV. While satisfaction rates with the vaccination process were high, caregivers had higher rates than their daughters (99.6% vs. 88.0%). The 11 Zimbabwe respondents who would not recommend the vaccine tended to have less education than Zimbabwe caregivers as a whole (10 with no more than primary school education).

## 4. Discussion

We found that integration of HPV vaccination into HIV services significantly increased the number of girls who received the vaccine post-integration compared with pre-integration, with more than a 30-fold increase in Mozambique. In Zimbabwe, HPV first dose coverage (97%) exceeded both the 80% national coverage and the WHO-recommended HPV vaccination coverage of 90%. After integration in Mozambique, we achieved HPV first dose coverage similar to the national program (68%). Higher coverage in Zimbabwe may be due to a more mature HPV program through a demonstration project (2014–2017) than the nationwide scale-up in 2018 compared to the national introduction in Mozambique in 2021 [9,11]. The AIM-HPV project in Zimbabwe also covered costs associated with outreach, making school and community-based vaccinations possible, while Mozambique fully leveraged existing platforms without additional cost inputs for services.

While some increase in HPV vaccination coverage could be expected with additional HCW training and community sensitization on HPV vaccination, the integration model was designed to improve coverage specifically for girls with HIV and at risk of HIV. Our study is one of the first to describe vaccination coverage disaggregated by girls’ HIV status. In Zimbabwe, the integration model effectively reached nearly all eligible GLHIV in study facilities with their first HPV dose, while Mozambique achieved 70% coverage in this population. The increases in this population are not likely to have been accomplished by a general increase in attention to HPV vaccination alone. Among GLHIV who were eligible for a second HPV dose by the end of the study, second dose coverage was lower in both countries at 74% and 49% in Zimbabwe and Mozambique, respectively, revealing some missed opportunities to fully vaccinate this population. For most eligible girls in both countries who did not receive a second dose, it was only two months past their second dose due date by the end of the study. Girls may have been on a longer multi-month ART dispensing schedule with fewer clinic visits (e.g., due to school), could have missed their visit entirely without successful follow-up, or ‘lost’ for vaccination if referred to another department without an escort or follow-up mechanism. However, these findings are consistent with previous two-dose policies for all girls which also showed a drop off in second dose coverage [25]. Moreover, in Zimbabwe, second-dose coverage is reported using a 12-month interval, which likely would have shown a higher completion rate [25].

This study was conducted following the shift in policy from a two-dose schedule for all girls to two doses only for GLHIV. Implementing effective delivery models that ensure GLHIV receive the second WHO-recommended dose is critical at a time when countries are grappling with these transitions to differential dose policies. HPV vaccination delivery approaches tend to rely on school-based or national campaigns [8,9,26] that do not typically ask for or assess HIV status to avoid threats to confidentiality and risk of stigmatizing children. The integration model addresses this challenge by ensuring that HPV vaccination is incorporated within HIV service requirements using the most appropriate model since providers already know the child’s status, have regular contact, and can integrate health education, vaccination, and active tracing of missed vaccine visits to existing service delivery. Even when GLHIV receive their first HPV vaccination with the general population through national delivery models, HIV providers should be responsible for asking and documenting receipt and ensuring that the girls receive their second dose on schedule. We found that there is not one implementation model that works best everywhere, but engaging stakeholders to identify organizational and patient characteristics and perspectives to inform the model of implementation best suited for each facility is critical. Ensuring that HCWs outside of the EPI program are knowledgeable about changing HPV vaccine strategies and documentation processes, particularly those providing HIV prevention and treatment services, is also key to seamless integration. While Tanzania introduced HPV vaccination through EPI, they similarly highlighted aspects such as the importance of delivery strategies tailored to individual HF and multisectoral collaboration and stakeholder trainings, as contributing to successful implementation [27].

In Mozambique, GLHIV were significantly more likely to receive their second dose at a co-location-only site compared to mixed or co-delivery sites. HIV providers at co-location-only sites targeted GLHIV and linked them with EPI for vaccination, placing less of a burden on HCWs (particularly compared to SAAJ clinics in other study HFs where girls with and without HIV were being vaccinated) and requiring fewer changes to clinic flow. The majority of these HFs were based in district headquarters, which are typically better resourced and more accessible to surrounding populations. Gaza Province was also associated with higher completion of the two-dose vaccination. There were more rural HFs in Gaza (which could suggest less accessibility) compared to Inhambane, and both provinces had the same procedures for identification and follow-up of vaccine-eligible girls. Therefore, differences may have been related to implementation, such as more integrated clinic flow and greater capacitation of HCWs for vaccination. For Zimbabwe, there were no statistically significant factors contributing to the receipt of a second dose.

Leveraging school-based platforms in Zimbabwe likely contributed to high vaccination coverage. Expansion of the vaccination model to include school settings at the request of girls and their caregivers eliminated the additional obstacle of traveling to clinics. Almost half of all dose 1 vaccinations took place in schools in Zimbabwe, though vaccination coverage for OOS girls was less than 1%. (It was not possible to capture this metric in Mozambique due to a reliance on routine records for HPV vaccination data.) This may be due in part because Zimbabwe has a relatively low rate of children out of primary school at 5% and an overall primary completion rate of 89%, though this varies by region [28]. Nonetheless, standard school-based vaccination can miss substantial numbers of girls not enrolled in formal education and OOS girls have socioeconomic vulnerabilities that put them at risk of HIV, HPV and later cervical cancer [29,30]. Tailored and multi-pronged strategies, such as complementary community-based and health service approaches, are needed to ensure this population of vaccine-eligible girls are identified and reached with HPV vaccination [25,26,29,30,31,32], which need to be coordinated with standardized procedures and reporting tools.

We found high vaccine acceptability and satisfaction with the delivery platforms under the integration model. Studies have reported high awareness and fear of cervical cancer and its perception as a serious disease contributed to HPV vaccine acceptability and motivated vaccine uptake [33,34,35]. In this study, more caregivers had heard of the HPV vaccine than had heard of HPV. Given that most caregivers knew about cervical cancer, there could be more of an association of the HPV vaccine with cancer than with HPV (despite it being in the name). Caregivers surveyed in Mozambique tended to have less knowledge on HPV and associated topics, were younger and less educated than caregivers from Zimbabwe. This could have played a role in vaccination uptake and underscores the importance of HPV vaccination demand creation and education that reaches caregivers at the time they are offered the vaccine [8]. For example, the availability of age-appropriate IEC materials targeted to specific audiences (e.g., girls with and without HIV, caregivers) for use in both facilities and communities could help to build awareness and address misinformation [27]. Caregivers of GLHIV may have had more awareness of HPV and vaccination due to their own HIV related care and cervical cancer risk.

The integration models included a network of community cadres leveraged for sensitization and demand creation. Peer leaders and CWs also mobilized girls in Zimbabwe, sometimes even going door-to-door to relay information or bring them to the HFs themselves. While they were not identified by caregivers as a common source of information on the HPV vaccine, nearly half of caregivers named CWs as a preferred messenger, particularly in Zimbabwe. With more formal training and support for their activities as part of a scale-up plan, such as reimbursement for airtime and transport within communities and to homes, they could play an even more prominent role in education, mobilization, and follow-up, reducing some of the HCW burden and promoting greater uptake, including among OOS girls [27,29,32,34,36]. Successful engagement would need to address potential challenges such as duplicative or incomplete documentation at the community level and equip CWs to effectively speak to concerns, misinformation or a lack of information on the vaccine.

There was only one caregiver identified who refused vaccination in the subset of 16 HFs and therefore the only one who participated in the survey, likely overestimating vaccine acceptability. While overall there were few vaccination refusals captured in chart abstractions, these too were underestimated as documentation of refusals, which was the responsibility of vaccine providers, was not consistently done. In Zimbabwe, refusals were underreported among caregivers interacting with community cadres or school health staff who did not document refusals. Relatedly, limited documentation of previous vaccination in routine records likely resulted in an underestimation of baseline vaccination coverage pre-integration, particularly in Mozambique. In Zimbabwe, a separate exercise was undertaken to address this issue, but a comprehensive review of all possible records was not feasible. The study found that accurate documentation of HPV vaccine access, acceptance and receipt across the multiple service delivery platforms is a challenge to address with close collaboration needed between the HIV and EPI teams in the MoH to ensure appropriate and integrated documentation of HPV vaccine needs, receipt, and follow-up for additional doses.

The intervention time period (due to funding end dates), approximately eight and nine months in Mozambique and Zimbabwe, respectively, may have underestimated the second dose coverage that would likely have been higher if there was a full year of intervention data. Nevertheless, our cut-off date was based on six or more months after the first dose, which was to be aligned with HIV appointments, so this also provides a marker of HIV integration feasibility. Despite these limitations, our study fulfilled a need for operational evidence on effective models of integrating HPV vaccination into other health services that are responsive to the needs of adolescents through the design and evaluation of an intervention which reached girls through HIV prevention and treatment services [37,38].

## 5. Conclusions

The AIM-HPV study demonstrated relatively high acceptability and effectiveness of the HPV integration model when targeted specifically to GLHIV and girls at risk of HIV acquisition, providing critical prevention for priority populations at greater risk for HPV infection and subsequent cervical cancer. This was achieved through coordination between the MoH HIV and EPI teams and design of multi-pronged approaches that centered vaccination delivery within HIV services (via co-location or co-delivery) for GLHIV. Scale-up of this approach will require improved integrated tools for individual-level eligibility screening and vaccination across entry points, particularly for GLHIV requiring multiple doses, and will require HPV vaccine receipt documentation in HIV care records that facilitate tracking for girls who are past due for a second dose. A sustainable HPV vaccination strategy will need to ensure activities external to the facility (e.g., community outreach) are appropriately budgeted for and that intervention models are adapted in each country to optimize reach of the most vulnerable girls, including those who are out of school.

## Figures and Tables

**Table 1:** Vaccination coverage by beneficiary group in Mozambique.

Beneficiary Group	Estimated # to Be Vaccinated	Doses Prior to Integration	1st Dose During Integration	2nd Dose During Integration		Total Vaccinated ≥1 Dose Prior to and During Integration	
		D1 only		D2 only	D1 and D2		
GLHIV, C&T	678	34 ^a^	195	30	36	229	69.6% (*n* = 472)
GLHIV, SAAJ		14	229	14	29	243	
HIV negative or unknown, SAAJ	2152	3 ^b^	1016	2	N/A	1019	63.5% (*n* = 1366)
DREAMS ^c^		3	344	3	N/A	347	
GLHIV, Co-location only	371	9	235	9	37	244	65.8%
Total	3201	63	2019	58 ^d^	102	2082	65.0%

GLHIV: Girls living with HIV; C&T: Care and Treatment; SAAJ: Serviços Amigos dos Adolescentes e Jovens, or Adolescent and Youth Friendly Services; DREAMS: Determined, Resilient, Empowered, AIDS-free, Mentored and Safe program; D1: Dose 1; D2: Dose 2; N/A: not applicable. ^a^ Four GLHIV received only one pre-integration dose; 30 received their second dose under integration; ^b^ One girl received only one pre-integration dose per policy; two received a second dose under integration; ^c^ Five DREAMS beneficiaries were living with HIV. Two of them received one dose pre-integration and one integration dose per policy; one other DREAMS beneficiary with HIV negative/unknown status received two HPV doses; ^d^ 55 GLHIV and three girls of HIV negative/unknown status received two doses. # Signifies number.

**Table 2:** Vaccination coverage by beneficiary group in Zimbabwe.

Beneficiary Group	Estimated # to Be Vaccinated	Vaccine Refusals	Doses Prior to Integration		1st Dose During Integration	2nd Dose During Integration		Total Vaccinated ≥1 Dose Prior to and During Integration
			D1	D1 and D2		D2 only	D1 and D2	
GLHIV, C&T	381	6	37	13	315	37	188	95.8% (*n* = 365)
DREAMS	1830	2	448	0	1302	367	1 ^a^	95.6% (*n* = 1750)
Orphans and Vulnerable Children	2223	3 ^b^	241	4	1924	48	0	97.6% (*n* = 2169)
Total	4434	11	726	17	3541	452	189	96.6% (*n* = 4284)

^a^ One girl with HIV; ^b^ One additional girl with unknown HIV status refused a second dose during integration but received one pre-integration dose and was therefore considered fully vaccinated. # Signifies number.

**Table 3:** Factors associated with receiving second HPV dose among eligible girls in Mozambique.

	Received Second HPV Dose		Model		
	No	Yes	Unadjusted	Adjusted	
	N (%)	N (%)	OR (95% CI)	OR (95% CI)	*p*-Value
Program beneficiary					
HIV clinic	88 (59.1)	61 (40.9)	1.0		
SAAJ	69 (62.7)	41 (37.3)	0.9 (0.4–2.0)		
Co-location only site					
No	111 (63.1)	65 (36.9)	1.0	1.0	
Yes	46 (55.4)	37 (44.6)	1.4 (0.5–3.5)	6.0 (1.9–19.0)	0.002 *
Child accompanied by caregiver for first dose					
No	15 (45.5)	18 (54.5)	1.0		
Yes	85 (63.9)	48 (36.1)	0.5 (0.2–1.5)		
Unknown	57 (61.3)	36 (38.7)	0.5 (0.2–1.8)		
Age group					
<10	30 (58.8)	21 (41.2)	1.0		
≥10	127 (61.1)	81 (38.9)	0.9 (0.4–1.9)		
Province					
Gaza	73 (51.0)	70 (49.0)	1.0	1.0	
Inhambane	84 (72.4)	32 (27.6)	0.4 (0.1–1.2)	0.1 (0.0–0.4)	0.001 *
HIV client volume (girls 9–14 years)					
5–14	11 (33.3)	22 (66.7)	1.0		
15–29	40 (56.3)	31 (43.7)	0.4 (0.1–2.1)		
30–49	61 (70.1)	26 (29.9)	0.2 (0.0–1.1)		
50–66	45 (66.2)	23 (33.8)	(0.0–1.9)		

* Significant at the 0.05 level.

**Table 4:** Demographics of children of caregiver survey participants eligible to receive the HPV vaccine.

Girls, N (%)	Mozambique (*n* = 299)		Zimbabwe (*n* = 401)	
	With HIV	Without HIV/unknown	With HIV	Without HIV/unknown
**Total N**	150	149	161	240
**Beneficiary**				
HIV clinic	120 (80.0)	N/A	156 (96.9)	N/A
SAAJ	25 (16.7)	110 (73.8)	N/A	N/A
DREAMS	5 (3.3)	39 (26.2)		135 (56.0)
OVC	N/A	N/A	5 (3.1)	105 (43.6)
**Girl age**				
9 years	33 (22.0)	11 (7.4)	17 (10.7)	19 (7.9)
10–14 years	117 (78.0)	138 (92.6)	142 (89.3)	221 (92.1)
*Missing*			*1*	*1*
**Caregiver relation**				
Biological mother	82 (54.7)	86 (57.7)	105 (65.6)	146 (60.6)
Biological father	7 (4.7)	1 (0.7)	7 (4.4)	6 (2.5)
Sibling	9 (6.0)	12 (8.1)	7 (4.4)	7 (2.5)
Other relative/friend	52 (34.6)	50 (33.5)	42 (26.3)	81 (33.6)
**Parents status**				
Both alive	107 (71.3)	137 (91.9)	103 (64.0)	171 (71.3)
Both died	0	0	10 (6.3)	3 (1.2)
1 parent died	41 (27.3)	10 (6.7)	47 (29.2)	64 (26.7)
Unknown	2 (1.3)	2 (1.3)	1 (0.6)	2 (0.8)
**Out of school**	6 (4.0)	4 (2.7)	8 (5.0)	0

**Table 5:** Caregiver knowledge of HPV, HPV vaccine and cervical cancer, and acceptability, experiences, and preferences of the vaccine and integration.

N (%)	Mozambique (*n* = 283)	Zimbabwe (*n* = 385)	Total (*n* = 668)
**Ever heard of cervical cancer**	210 (74.2)	364 (94.5)	574 (85.9)
**Know someone with cervical cancer**	72 (34.3)	131 (36.0)	203 (35.4)
**Ever heard of HPV**	133 (45.1)	249 (64.7)	382 (56.2)
**What have you heard?** (most common responses) ^a^	N = 133	N = 249	N = 382
HPV can cause cervical cancer in women	62 (46.6)	176 (70.7)	238 (62.3)
HPV is transmitted through sex	51 (38.3)	108 (43.4)	159 (41.6)
Only women can get infected with HPV	82 (61.7)	62 (24.9)	144 (37.7)
**Ever heard of HPV vaccine**	141 (49.8)	316 (82.1)	461 (67.8)
**What have you heard?** (most common responses) ^a^	N = 141	N = 316	N = 457
HPV vaccine protects against cervical cancer	96 (68.1)	280 (88.6)	376 (82.3)
HPV vaccine protects against cancer, not sure which type	41 (29.1)	25 (7.9)	66 (14.4)
Don’t know	8 (5.7)	9 (2.8)	17 (3.7)
**Most common concerns with HPV vaccine ^a^**	N = 141	N = 316	N = 457
None	113 (80.1)	277 (87.7)	390 (85.3)
Affects fertility	9 (6.4)	18 (5.7)	27 (5.9)
Makes you feel a bit sick after	13 (9.2)	10 (3.2)	23 (5.0)
Painful injection	4 (2.8)	12 (3.8)	16 (3.5)
**Most common sources of information on HPV vaccine ^a^**	N = 141	N = 316	N = 457
Group health education at health facility (HF)	64 (45.4)	152 (48.6)	216 (47.6)
Radio	13 (9.2)	158 (50.5)	171 (37.7)
Individual counseling with healthcare worker (HCW)	36 (25.5)	61 (19.5)	97 (21.4)
Care and treatment support group	34 (24.1)	46 (14.7)	80 (17.6)
TV	28 (19.9)	43 (13.7)	71 (15.6)
*Missing*	0	3	3
**Most common preferences for HPV vaccination information sources ^a^**	N = 283	N = 385	N = 668
HCW	226 (80.1)	360 (93.5)	586 (87.9)
Community worker	61 (21.6)	242 (62.9)	303 (45.4)
No preference	54 (19.1)	17 (4.4)	71 (10.6)
*Missing*	1	0	1
**Accepted HPV for ≥1 eligible girls**	283 (100.0)	384 (99.7)	667 (99.9)
**Most common reasons for accepting vaccine ^a^**	N = 283	N = 384	N = 667
Believed vaccination would protect against HPV/cancer	230 (83.0)	287 (75.1)	517 (78.5)
HCW encouraged vaccination	103 (37.2)	43 (11.3)	146 (22.2)
Trusted the safety/quality of the vaccine	82 (29.6)	46 (12.0)	128 (19.4)
*Missing*	6	2	8
**Satisfaction with daughter’s HPV vaccination in this setting (e.g., DREAMS/OVC, HIV C&T)**	N = 283	N = 384	N = 667
Not at all satisfied	1 (0.4)	2 (0.5)	3 (0.5)
Somewhat satisfied	23 (8.4)	82 (21.5)	105 (16.0)
Very satisfied	251 (91.3)	298 (78.0)	549 (83.6)
*Missing*	8	2	10
**Daughter’s satisfaction with HPV vaccination in this setting**	N = 283	N = 384	N = 667
Very satisfied	166 (60.4)	217 (56.7)	383 (58.2)
Somewhat satisfied	84 (30.5)	112 (29.2)	196 (29.8)
Not at all satisfied	8 (2.9)	8 (2.1)	16 (2.4)
Don’t know	17 (6.2)	46 (12.0)	63 (9.6)
*Missing*	8	1	9
**Setting preference for daughter’s receipt of HPV vaccine**	N = 283	N = 385	N = 668
HF as part of receiving other services	113 (40.9)	207 (54.0)	320 (48.6)
HF for immunization only	122 (44.2)	76 (19.8)	198 (30.0)
No preference	33 (12.0)	37 (9.7)	70 (10.6)
School	2 (0.7)	52 (13.6)	54 (8.2)
Community locations or social events	5 (1.8)	10 (2.6)	15 (2.3)
Did not want her vaccinated at all	1 (0.4)	0	1 (0.2)
Unsure	0	1 (0.3)	1 (0.2)
*Missing*	7	2	9
**Likelihood you would recommend HPV vaccine**	N = 283	N = 385	N = 668
Very likely	204 (72.1)	270 (70.1)	474 (71.0)
Likely	77 (27.2)	104 (27.0)	181 (27.1)
Not at all likely	2 (0.7)	11 (2.9)	13 (1.9)

^a^ Could select more than one response.

## Data Availability

The raw data supporting the conclusions of this article will be made available by the authors on request.

## Supplementary Materials

The following supporting information can be downloaded at: https://www.mdpi.com/article/10.3390/vaccines14060503/s1, Table S1: Factors associated with receiving the second HPV dose among eligible girls in Zimbabwe; Table S2: Selected caregiver survey data by HIV status of child.

## Abbreviations

The following abbreviations are used in this manuscript:

ART	Antiretroviral therapy
C&T	Care and treatment
CW	Community worker
DREAMS	Determined, Resilient, Empowered, AIDS-free, Mentored and Safe
EPI	Expanded Programme on Immunization
FCH	Family and Child Health
GLHIV	Girls living with HIV
HF	Health facility
HPV	Human Papillomavirus
HCW	Healthcare worker
IEC	Information, education, and communication
INS	Instituto Nacional de Saúde
IQR	Interquartile range
MoH	Ministry of Health
OOS	Out of school
OPD	Outpatient department
OVC	Orphans and Vulnerable Children
PEPFAR	President’s Emergency Plan for AIDS Relief
PRISM	Practical, Robust Implementation and Sustainability Model
RA	Research assistant
RE-AIM	Reach, Effectiveness, Adoption, Implementation, and Maintenance
SAAJ	Serviços Amigos dos Adolescentes e Jovens
STI	Sexually transmitted infection
SWO	Stop work order
WHO	World Health Organization
