# Supplementary material for: HPV Vaccination Uptake and Acceptability of HPV/HIV Integrated Services Models for Adolescent Girls in Mozambique and Zimbabwe: The AIM-HPV Implementation Research Study
Source: Vaccines (Basel). 2026 Jun 3;14(6):503. doi: 10.3390/vaccines14060503 (PMC13307793; doi:10.3390/vaccines14060503)
Supplement: Supplementary file 1 [file vaccines-14-00503-s001.zip › Supplemental Table S1.pdf]

Supplemental Table S1. Factors associated with receiving the second HPV dose among eligible girls in Zimbabwe

	Received Second HPV dose		Model
	No	Yes	Unadjusted
	N (%)	N (%)	OR (95% CI)
Facility level			
Clinic	38 (33.9)	74 (66.1)	1.0
Hospital	12 (25.0)	36 (75.0)	1.5 (0.4 - 6.1)
Polyclinic	37 (31.9)	79 (68.1)	1.1 (0.6 - 2.1)
First dose location			
Health Facility	75 (29.4)	180 (70.6)	1.0
Community	6 (40.0)	9 (60.0)	0.6 (0.2 - 1.7)
Child in school			
No	1 (20.0)	4 (80.0)	1.0
Yes	85 (32.3)	178 (67.7)	0.5 (0.1 - 2.4)
Age group			
<10	12 (41.4)	17 (58.6)	1.0
≥ 10	75 (30.4)	172 (69.6)	1.6 (0.7 - 3.6)
District			
Mazowe	31 (33.0)	63 (67.0)	1.0
Harare	56 (30.8)	126 (69.2)	1.1 (0.5 - 2.5)
