# Supplementary material for: HPV Vaccination Uptake and Acceptability of HPV/HIV Integrated Services Models for Adolescent Girls in Mozambique and Zimbabwe: The AIM-HPV Implementation Research Study
Source: Vaccines (Basel). 2026 Jun 3;14(6):503. doi: 10.3390/vaccines14060503 (PMC13307793; doi:10.3390/vaccines14060503)
Supplement: Supplementary file 1 [file vaccines-14-00503-s001.zip › Supplemental Table S2.pdf]

Supplemental Table S2. Selected □aregiver survey data by HIV status of child

N (%)	Mozambique		Zimbabwe	
**Child HIV status**	**All without HIV or unknown status**	**≥1 child with HIV**	**All without HIV or unknown status**	**≥1 child with HIV**
**N**	141 (49.8)	142 (50.2)	224 (58.2)	161 (41.8)
**Caregiver education**				
None	6 (4.3)	29 (20.4)	10 (4.5)	5 (3.1)
Some primary	67 (47.5)	83 (58.5)	34 (15.2)	11 (6.8)
Primary complete	47 (33.3)	21 (14.8)	75 (33.5)	49 (30.4)
Secondary or higher	21 (14.9)	9 (6.3)	105 (46.9)	96 (59.6)
**Caregiver HIV status**				
Living with HIV	28 (19.9)	92 (64.8)	41 (18.3)	123 (76.4)
**Heard of cervical cancer before?**	94 (66.7)	116 (81.7)	215 (96.0)	149 (92.5)
**Of those who have heard of cervical cancer, know anyone who has had cervical cancer?**	32 (34.0)	40 (34.5)	64 (29.8)	67 (45.0)
**Heard of HPV before?**	58 (41.1)	72 (50.7)	131 (58.5)	118 (73.3)
**What have you heard?** [a]	**N=58**	**N=72**	**N=131**	**N=118**
Only women can get infected with HPV	32 (55.2)	50 (69.4)	28 (21.4)	34 (28.8)
Women and men can get HPV	17 (29.3)	15 (20.8)	12 (9.2)	19 (16.1)
HPV can cause cervical cancer in women	16 (27.6)	46 (63.9)	87 (66.4)	89 (75.4)
HPV can cause genital warts	18 (31.0)	31 (43.1)	9 (6.9)	21 (17.8)
A person could have HPV for many years without knowing it	4 (6.9)	20 (27.8)	18 (13.7)	11 (9.3)
HPV is transmitted through sex	13 (22.4)	36 (50.0)	43 (32.8)	65 (55.1)
Nearly all sexually active people will get HPV at some point	3 (5.2)	2 (2.8)	27 (20.6)	23 (19.5)
The chance of getting HPV increases with the number of sexual partners	1 (1.7)	6 (8.3)	32 (24.4)	14 (11.9)
**Heard about the HPV vaccine before?**	65 (46.1)	76 (53.5)	177 (79.0)	139 (86.3)
**What have you heard?** [a]				

N (%)	Mozambique		Zimbabwe	
**Child HIV status**	**All without HIV or unknown status**	**≥1 child with HIV**	**All without HIV or unknown status**	**≥1 child with HIV**
HPV vaccine protects against cancer, not sure which type	13 (20.0)	28 (36.8)	11 (6.2)	25 (7.9)
HPV vaccine protects against cervical cancer	48 (73.8)	48 (63.2)	155 (87.6)	280 (88.6)
HPV vaccine protects against genital warts	7 (10.8)	20 (26.3)	1 (0.6)	10 (7.2)
HPV vaccine protects against sexually transmitted infection	1 (1.5)	17 (22.4)	2 (1.1)	10 (7.2)
**No concerns with vaccine**	52 (80.0)	61 (80.3)	154 (87.0)	123 (88.5)
**Preferred source of information on HPV vaccine[a]**	**N=141**	**N=142**	**N=224**	**N=161**
Healthcare worker	114 (80.9)	112 (79.4)	207 (92.4)	151 (93.8)
Community health worker (CHW, VHW, Mentor Mother)	27 (19.1)	34 (24.1)	151 (67.4)	91 (56.5)
Missing	*0*	*1*	*0*	*0*
**Preferred place to get HPV information[a]**	**N=141**	**N=142**	**N=224**	**N=161**
Group health education - in community	38 (27.0)	40 (28.2)	73 (33.2)	40 (25.5)
Group health education - at facility	87 (61.7)	90 (63.4)	166 (75.5)	129 (82.2)
Radio	20 (14.2)	10 (7.0)	57 (25.9)	78 (49.7)
TV	33 (23.4)	25 (17.6)	16 (7.3)	45 (28.7)
Missing	*0*	*0*	*4*	*4*
**Did daughter receive HPV information?**				
No	36 (25.5)	17 (12.0)	17 (7.6)	17 (10.6)
Yes	81 (57.4)	120 (84.5)	158 (70.5)	123 (76.4)
Not sure	24 (17.0)	5 (3.5)	49 (21.9)	21 (13.0)

[a] Could select more than one response; most common responses only listed
